# The effect of an interactive weekly mobile phone messaging on retention in prevention of mother to child transmission (PMTCT) of HIV program: study protocol for a randomized controlled trial (WELTEL PMTCT)

**DOI:** 10.1186/s12911-016-0321-4

**Published:** 2016-07-11

**Authors:** Patricia Opondo Awiti, Alessandra Grotta, Mia van der Kop, John Dusabe, Anna Thorson, Jonathan Mwangi, Rino Belloco, Richard Lester, Laura Ternent, Edwin Were, Anna Mia Ekström

**Affiliations:** Department of Public Health Sciences, Karolinska Institutet, 171 77 Stockholm, Sweden; Department of Medical Epidemiology and Statistics, Karolinska Institutet, 171 77 Stockholm, Sweden; Division of Infectious diseases, University of British Columbia, Vancouver, Canada; Division of Global Health, University of British Columbia, Vancouver, Canada; Department of Public Health Sciences, 171 77 Stockholm, Sweden; Department of Medicine, University of British Columbia, Vancouver, Canada; Newcastle University, Tyne and Wear, NE1 7RU UK; Department of Reproductive Health, Moi University, P.O.BOX 3900–30100, Eldoret, Kenya

**Keywords:** Mobile health (m-health), Retention, HIV/PMTCT, Antiretroviral therapy (ART), Kenya

## Abstract

**Background:**

Improving retention in prevention of mother to child transmission (PMTCT) of HIV programs is critical to optimize maternal and infant health outcomes, especially now that lifelong treatment is immediate regardless of CD4 cell count). The WelTel strategy of using weekly short message service (SMS) to engage patients in care in Kenya, where mobile coverage even in poor areas is widespread has been shown to improve adherence to antiretroviral therapy (ART) and viral load suppression among those on ART. The aim of this study is to determine the effect of the WelTel SMS intervention compared to standard care on retention in PMTCT program in Kenya.

**Methods:**

WelTel PMTCT is a four to seven-centers, two-arm open randomized controlled trial (RCT) that will be conducted in urban and rural Kenya. Over 36 months, we plan to recruit 600 pregnant women at their first antenatal care visit and follow the mother-infant pair until they are discharged from the PMTCT program (when infant is aged 24 months). Participants will be randomly allocated to the intervention or control arm (standard care) at a 1:1 ratio. Intervention arm participants will receive an interactive weekly SMS ‘How are you?’ to which they are supposed to respond within 24 h. Depending on the response (ok, problem or no answer), a PMTCT nurse will follow-up and triage any problems that are identified.

The primary outcome will be retention in care defined as the proportion of mother-infant pairs coming for infant HIV testing at 24 months from delivery. Secondary outcomes include a) adherence to WelTel; (b) adherence to antiretroviral medicine; (c) acceptance of WelTel and (d) cost-effectiveness of the WelTel intervention.

**Discussion:**

This trial will provide evidence on the effectiveness of mHealth for PMTCT retention. Trial results and the cost-effectiveness evaluation will be used to inform policy and potential scale-up of mHealth among mothers living with HIV.

**Trial registration:**

ISRCTN98818734; registered on 9th December 2014

## Background

### HIV and maternal and newborn health in Kenya

Identification and rollout of the most cost-effective interventions to eliminate HIV infections and AIDS death among women and children is a key priority for Kenya. According to the AIDS Response Progress Report 2014, the number of people living with HIV (PLHIV) in Kenya has increased by 15 % over the last 4 years, reaching 1.6 million cases in 2013, with an estimated incidence of100 000 new HIV infections in 2014 [1]. Women represent about 57 % of all PLHIV in Kenya and close to 1 % (101,000) of all Kenyan children are living with HIV, corresponding to 10 % of all PLHIV [1]. As many as 11 000 Kenyan children were estimated to be newly infected in 2013, mainly through mother to child transmission (MTCT). Thus, Kenya’s commitment to eliminate MTCT of HIV by 2015 will not be achieved, but improvements have been made. Over the last decade, the number of HIV-infected pregnant women in need for PMTCT in Kenya has also declined, albeit too slowly, from 98,000 in 2004 to 79,000 in 2013 [1]. Since 2013, Kenya has adopted PMTCT Option B+, the World Health Organization guideline recommending all pregnant women living with HIV receive immediate HIV treatment *for life* regardless of immune defense (CD4 count), a strategy called PMTCT Option B+ [2].

Antiretrovirals (ARVs) during pregnancy, delivery and breastfeeding, and for the infant 6 weeks post-delivery can reduce the risk of transmission from 35 % to <2 % in low-income countries [3]. Key strategies to finally eliminate MTCT include increased knowledge of PMTCT, increased involvement from the male partner, universal attendance of antenatal care (ANC) by pregnant women, universal testing of pregnant women for HIV and provision of ARVs from early pregnancy throughout the breastfeeding period, and facility delivery [4]. Kenya’s progress on these goals is uneven. While the proportion of pregnant women tested for HIV has increased from 68 to 92 % in the last 5 years [1], only 5 % of male partners accompanied their pregnant partner to ANC [1]. PMTCT coverage (the number of pregnant women living with HIV started on ARVs before delivery) declined in Kenya from 86 % in 2010 to 73 % in 2013, i.e. 58,000 out of 79,000 pregnant women living with HIV were offered PMTCT services [1]. This was partly due to the multiple challenges of implementing Option B+, which is much more resource demanding.

Only 50 % in need of PMTCT were given ARVs within 6 weeks of their HIV diagnosis [1], a service delay that may cause avoidable MTCT. Only 45 % of all HIV-exposed infants were tested for HIV, i.e. the majority of children were lost to follow up leading to preventable child deaths [1]. Because of these shortcomings, the proportion of HIV-exposed children who became HIV-infected has halted at 14 % in the last 3 years. If Kenya is to achieve the global target of eliminating MTCT of HIV, initially aimed for 2015, this trend needs to be reversed through new and more effective interventions.

### Mobile phone use for information and health service strengthening in Kenya

The rapid expansion in mobile phone technology in Africa has created new opportunities for information sharing and service delivery where other infrastructure, such as cable connectivity and constant electricity supply is inadequate. In 2013, 82 % of Kenyan households owned a mobile phone [5], reaching 100 % of 20–29 year-olds i.e. the age-period when most women give birth [6]. In fact, Kenya has the world’s highest proportion of cell phone owners (80 %) who use mobile banking, and 60 % of Kenyans living on less than $2,50 i.e. under the poverty line, have mobile phones [6].

Mobile technology for health (mHealth) is increasingly being used to overcome shortcomings in information systems, laboratory equipment and human resource capacity in low-income countries. Within maternal and child health care (MCH), mobile technology has been used to link ANC with pregnant women and new mothers [7], to remind community health workers in rural areas [8] and to submit surveillance reports on disease outbreaks and delivery of services [9]. Other uses of mHealth include registration of records [10] and monitoring of drug procurements [11, 12].

Several trials have highlighted the potential of mHealth to improve HIV services e.g. in adherence to ART, retention in ART care, and even for receipt of laboratory HIV test results [13–15]. A recent Cochrane review, which included the WelTel RCT in Kenya found compelling evidence that weekly text messages to non-pregnant HIV infected patients are effective [16] in improving ART adherence.

Whether a weekly interactive SMS intervention (the WelTel model) improves adherence and retention in PMTCT care and encourages life-long ARVs is unknown. The situation for pregnant women living with HIV in a country like Kenya is often highly complicated. Many women do not feel comfortable disclosing their HIV status to a partner or family members in fear of being stigmatized and socially isolated [17], which greatly influences adherence to ART and PMTCT. Furthermore, most women are newly diagnosed with HIV during pregnancy, often asymptomatic and have little time to adjust to the idea of living with HIV before they must start on ARVs. Our previous research shows that competing burdens, including breadwinning responsibilities, stigma, feelings of guilt and fear of transmitting HIV to the baby, fear of abandonment and violence from the male partner also affects women’s capacity to adhere to PMTCT [18].

The WelTel trial remains the only SMS adherence intervention study with the lowest risk of bias ranking. The WelTel service has other advantages in that it is low cost since it uses SMS to check-in on patients, can work with minimal literacy since problems can be followed up with voice calls and extends into the population that do not own their own phones since shared access is sufficient, and by being open-ended check-ins allows almost any problem to be triaged. We will assess the effectiveness, including evaluation of costs of weekly interactive SMS reminders to improve the retention of pregnant women and mother-infant pairs in PMTCT care in Kenya. Ultimately this will result in better PMTCT coverage and reduced infant HIV infections.

### Research hypothesis

The weekly interactive mobile phone SMS (WelTel) is an effective as well as cost-effective method to improve the retention of women living with HIV and their newborns’ in PMTCT care (women living with HIV and their HIV exposed infants who successfully complete the program when infant is aged 24 months).

### Study objectives

#### Primary objective

To determine effectiveness of the WelTel SMS intervention on retention of women living with HIV and their newborns’ in PMTCT care in urban and rural Kenya.

#### Secondary objectives

1. To assess adherence to the WelTel SMS intervention among pregnant women and newly delivered mothers living with HIV.
2. To determine adherence to single components of PMTCT among pregnant women and newly delivered mothers living with HIV (ARVs, facility-based delivery, early infant HIV testing and exclusive breastfeeding).
3. To explore facilitators for and barriers to using WelTel SMS in order to inform any improvements on the model for PMTCT among pregnant women and newly delivered mothers living with HIV as well as PMTCT staff.
4. To evaluate costs from a payer’s perspective, of the WelTel SMS for retaining women living with HIV and HIV-exposed infants in clinical follow-up until 24 months post-delivery (discharge from PMTCT).

## Methods/design

### Trial design

**Fig. 1:**
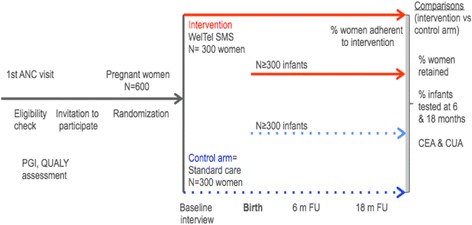
WelTel PMTCT trial design

The WelTel PMCT study is a 4–7 center two-arm open randomized controlled trial in which the intervention is allocated in a 1:1 ratio (Fig. 1 trial design).

### Study setting

The study is in western Kenya and involves 4–7 facilities that are among over 192 facilities providing PMTCT services located in the catchment of Academic Model Providing Access to Health Care (AMPATH) – a large HIV Comprehensive Care Program run under the auspices of Moi University School of Medicine, located in Eldoret. These facilities implement Option B+ regimen of PMTCT. The approximate coverage of mobile phones in western Kenya is around 78 % [19]. The research setting has been carefully selected to represent urban and rural mixes that have high antenatal HIV percentage prevalence (10–15 %), about twice that of the national prevalence of 6 %.

### Study population

The study population will consist of: (i) pregnant women living with HIV aged 18 and over presenting at ANC for a first visit in the current pregnancy at the selected clinics; and (ii) newborns delivered to these women living with HIV. Women who will be pregnant and will be diagnosed with HIV infection will be referred to a male or female research assistant to complete a checklist for eligibility. Determination of HIV infection will be based on two repeated Determine or Colloidal Gold tests for women newly diagnosed during the current pregnancy, or, based on referral from the comprehensive care clinic for those with known HIV infection and on antiretroviral therapy (ART) or pre-ART). Individuals must fulfill all the inclusion criteria, provide consent to participate and complete an interviewer-administered questionnaire before they are randomized to either the control and intervention groups.

#### Inclusion criteria

1. Women aged 18 years or above
2. Evidence of pregnancy
3. Evidence of HIV infection
4. Resident of the PMTCT clinic catchment area and plans to remain residents from recruitment until 24 months after delivery
5. Willing to be followed-up from recruitment until 24 months after delivery
6. Owning a mobile phone or having access to a mobile phone
7. Able to text message in Kiswahili or have someone in close contact that they trust to read and respond to a text message
8. Willing to receive text messages from the PMTCT clinic staff
9. Able and willing to provide informed consent

### Intervention

**Fig. 2:**
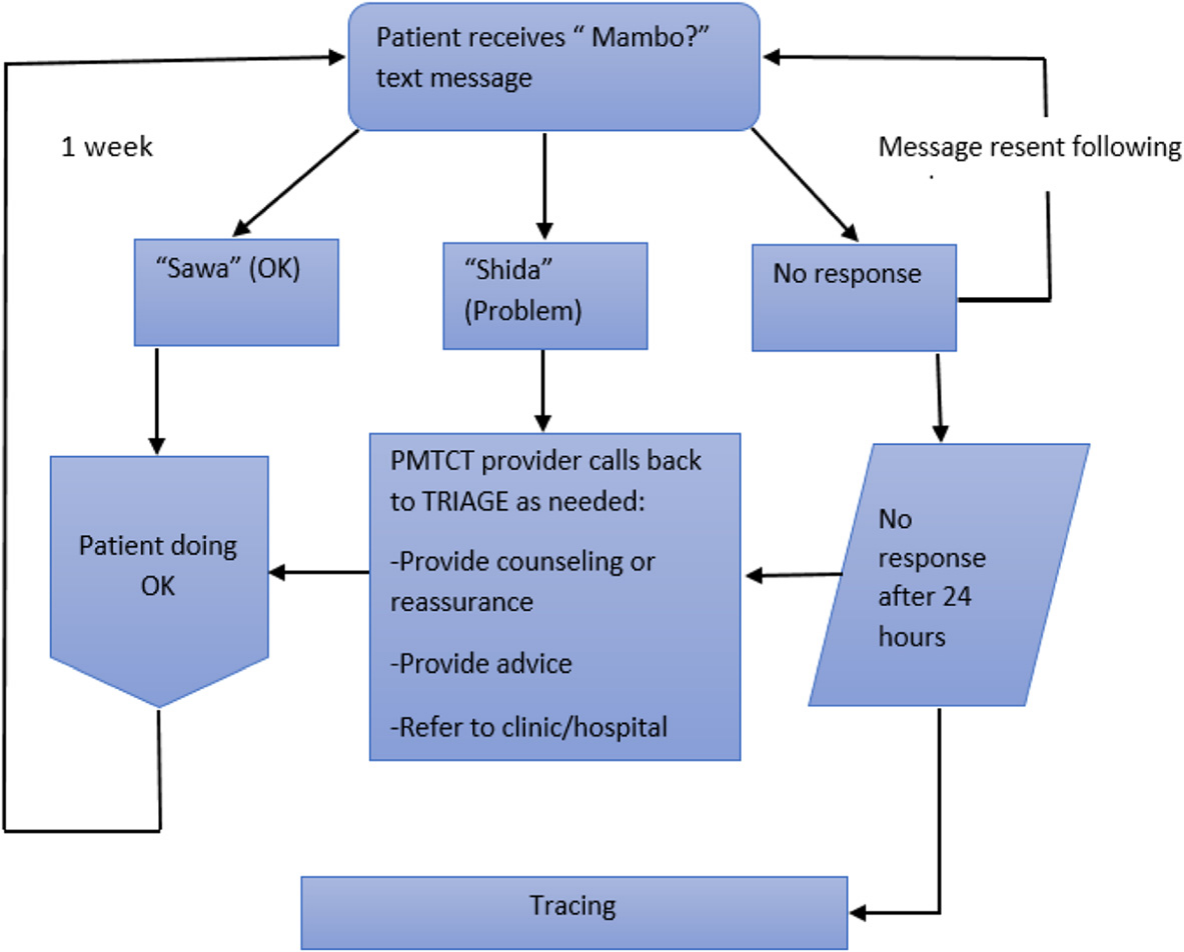
WelTel SMS intervention

Participants in the intervention group will register their phone numbers in the WelTel system (online or via SMS) and then receive a weekly short text message question in Kiswahili “*Mambo?”* (Kiswahili for “How are you?”) asking about their general wellbeing (Fig. 2). The message will be sent on a fixed day of the week and will allow the patient to respond within 24 h either that they are well for example “*ok”* or “*sawa”* or that they have a problem (for example “problem*”* or “*shida”).* A female study coordinator will be in charge of centrally monitoring the WelTel SMS platform, which automatically sends the messages and registers responses from the participants and categorizes them. All participants who respond “problem” or who do not respond will be directly linked to a regular PMTCT nurse at the woman’s clinic to assist with identified problems. Problems that cannot be immediately resolved by the nurse follow routine procedures at the clinic and are normally referred to the PMTCT clinical officer at the respective facility who will then decide if the patient needs to visit the facility or should receive a follow up phone call. The study coordinator will follow up with the respective PMTCT nurses to record action taken, which is entered directly into the WelTel platform logs as notes. Patients who will not respond to the SMS within 24 h will be traced (first by telephone then at households) within the defaulter tracing outreach program in routine PMTCT care. At enrollment, the participants will be informed that the weekly SMS support service does not replace routine clinic services, and that all appointments made by PMTCT staff should be honored and all emergencies should be handled by usual means. A WelTel SMS platform technician will handle all technical problems that may arise.

### Outcomes

#### Primary outcome

Retention in PMTCT care is defined as the proportion of women living with HIV and their HIV-exposed infants that remain in care until infants are aged 24 months measured from when the pair is enrolled in the program from the woman’s first visit at ANC until 24 months after birth.

#### Secondary outcomes

1. Adherence to the WelTel SMS intervention measured as the proportion of women living with HIV who do not respond to the SMS within 7 days.
2. Adherence to (i) ARVs measured as the proportion of women living with HIV who do not respond to the SMS within 7 days when suspected to be out of ARVs (i.e. failure to pick up ARVs that can cover her absence); (ii) facility-based delivery measured as the proportion of women who deliver in hospital; (iii) early infant HIV testing defined as the proportion of HIV-exposed infants who are tested for HIV within 8 weeks of birth measured as HIV –exposed infants with known HIV status at age 10weeks and (iii) exclusive breastfeeding defines as feeding the HIV-exposed infant only milk from the mother’s breast.
3. Participant perceived facilitators and barriers of the WelTel SMS intervention (assessed as perceived reasons and challenges of use as well as suggestions to possible solutions).
4. Cost compared with (i) effectiveness (levels of retention) from a payers’ perspective and (ii) quality of life (differences in pregnant women’s QALYs between the two trial arms.

### Sample size

The primary outcome of the study is defined as the proportion of mother-newborn retained in PMTCT care at 24 months; assuming i) a power of 80 %, ii) a two-sided test (alpha = 0.05), iii) and based on prior knowledge, a proportion retained in the control group of about 30 %, a sample size of 300 participants in each arm for a total of 600 subjects, with 5 % dropout rate (i.e. women who decide to withdraw from the study) in both the control and intervention arms was estimated to detect a 11 % difference (the smallest detectable difference) in the primary outcome between the intervention and control arms that is the difference that it would be important to detect is 11 %, computed as the proportion retained in the intervention arm minus the proportion retained in the control arm i.e. the proportion retained in the intervention group minus the proportion retained in the control group. Calculations were performed using Stata 14.1.

### Recruitment

A PMTCT nurse at the selected clinics will inform all consecutive pregnant women identified as living with HIV at their first ANC visit about the study. The PMTCT nurse will then refer these clients to the research assistant who will assess their eligibility and provide detailed information about the study. Individuals who are eligible will be invited to participate in the study and the research assistant will seek their consent.

Participants in the intervention arm who own or have access to mobile phones will be registered directly onto the WelTel platform with their phone number.

### Randomization and allocation

Eligible and consenting patients will be randomized to the intervention and control arms using a 1:1 allocation ratio. To ensure balance between the arms throughout the trial, we adopted a permuted-block randomization scheme. The block size will be concealed until the trial is over. Randomization will be performed separately at each clinic. We will use opaque sealed envelopes to assign participants to the intervention and control arms. The randomization list was be generated at the Karolinska Institutet (Stockholm, Sweden) by an independent statistician.

### Baseline

An interviewer- facilitated baseline questionnaire will be administered after recruitment and allocation. Questions record information on participants’ social and demographic characteristics; time of HIV diagnosis, time on ARV, disclosure of HIV status, HIV care and social support, mobile phone use as well as costs for accessing care.

### Follow up

Follow up visits will occur at 6 and 24 months postpartum, at which time research assistant will administer the follow-up questionnaire. The follow up questionnaire will capture information on participants’ missed appointments, engagement with health workers and satisfaction with care, mobile phone access and using the WelTel intervention, and health related status/quality of life using the EuroQol 5-dimensional (EQ-5D) utility scores and twelve item short form survey (SF-12) standardized tools.

To investigate quality of life, we will first develop a patient generated index (PGI) to identify areas in the women’s lives that are affected by their HIV infection during pregnancy and nursing period; periods when the risk of transmitting HIV is high. A PGI is an individualized patient reported instrument that allows the respondent to state, weight and rate areas of importance to the patients’ lives that are affected by their illness. The PGI will also enable the investigators to assess the validity of the EQ5D and SF12 in this population and context.

### Data collection and management

All outgoing and incoming text messages will be automatically recorded on the WelTel platform. The platform also captures all of the “problems” noted by participants, instances of non-response, and actions taken in relation to participants’ ‘problem’ responses and non-responses. Platform data will be backed up every 7 days.

All questionnaires will be paper-based and the data manager will then enter data into a database at the central office on an ongoing basis. A data manager will check the forms for completeness and quality will be verified by re-checking a random sample of 10 % of the data. Any problems that arise will be resolved promptly. Participant files will be stored in a locked office at the trial sites.

Data on attendance, HIV care clinical indicators like viral load and CD4 cell count as well as treatment regimen will be collected medical records. We will also collect information on demographic characteristics (age, education level, marital status and parity) of all screened patients/potential trial participants.

Qualitative research using in –depth interviews will also be performed to discover ‘how’ and ‘why’ the intervention works to improve retention in PMTCT program. Purposive sampling will be used to identify participants for qualitative interviews. Face-to-face interactions will be used to build trust during the interview process and to enhance free interaction between the researcher and the participants [20]. All conversations will be recorded with permission from the respondents and the interviews will be performed at a place and in a language preferred by the respondent.

All qualitative research tools will be developed in English, then translated to Kiswahili, and then back translated to English. The interview guide will be available in both languages.

### Analysis

#### Statistical methods

Baseline participants’ characteristics will be reported separately by treatment arm. Baseline characteristics include: age, parity (nulliparous versus previous birth), marital status (single, married or cohabiting, divorced/widowed), ARV exposure (experienced vs. naive), duration of known HIV diagnosis (newly vs. previously diagnosed), age (18–29, 30–39, 40–49, ≥50 years of age), phone ownership (owned vs. shared), level of education (none, primary, secondary, post-secondary), distance from clinic (≤1 h vs. >1 h), number of children born after HIV diagnosis, on ARV at enrolment (yes, no), time on ARV at enrolment (≤6 months, 7–12 months, ≥13 months) and HIV status disclosure (yes, no). We will report the mean (standard deviation [SD]) or median (first quartile, third quartile) for continuous variables, and count and percentages for categorical variables. All analyses are by *intention-to-treat* i.e. according to the study group to which women were originally allocated regardless of subsequent intervention received and per protocol. Other more statistical methods will be used to take into account switching.

For the primary outcome, we will compare the proportion of mothers living with HIV and their HIV-exposed infants in the program at 24 months post-birth in the intervention vs. control arm using both parametric (Chi-Square) and exact (Fisher) statistical tests. Secondary outcomes will also be compared between arms, with t-tests for normally distributed variables, Mann Whitney-U tests for non-normally distributed variables, and Chi-Square and Fisher exact tests for categorical variables. The relative risk (RR) for PMTCT retention with 95 % confidence intervals will be computed and the number needed to treat to prevent one non-retained mother-infant pair will also be estimated. Concerning the secondary outcomes, average treatment effects (ATE) will be computed for continuous outcomes and RRs for categorical outcomes. For both primary and secondary outcomes, log-linear or linear regression models will be used to provide effect estimates adjusted for potential imbalances in baseline participants’ characteristics, if required. We will repeat the analysis of primary and secondary outcomes within such subgroups (in relation to socio-demographics) to assess the homogeneity of the intervention effect across pre-determined subgroups of patients. Stratified RRs and ATEs will be computed. Regression models, which include the intervention allocation and subgroup-defining variables and their interaction, will be applied to assess effect modification across groups; all statistics tests will be run based on two side *p*-values and values <0.05 will be considered statistically significant. All statistical analyses will be again performed with Stata version 14.1 (Stata Corporation, College Station, TX, USA).

#### Qualitative process evaluation

Qualitative data will be analyzed using content analysis, guided by Graneheim and Lundman [21]. First, the transcribed material is read a number of times to get a general sense of the material by a group of researchers. Using the open code software for qualitative research, meaning units, which are key phrases in the text, are identified, condensed and outlined. Codes will then be ascribed to each meaning unit. The codes will be compared and grouped into sub-categories. This comparison will be performed consistently to identify emerging categories that will be further compared, re-organized and merged into sub-themes and one overarching theme. The coding and analysis process will be deductive in nature and involve key members of the research team.

### Economic evaluation

We will perform an economic evaluation to assess WelTel SMS from a healthcare payer perspective. The primary outcome of the cost effective analysis (CEA) will be the incremental cost per additional mother-infant pairs that remain in PMTCT until infant is aged 24 months. A secondary outcome is averted infant HIV-infections at cessation of breastfeeding. For the cost-utility analysis the outcome will be quality adjusted life years (QALYs), based on responses to the SF-12 and EQ5D. For both analyses (CEA and CUA) we will report the incremental cost-effectiveness ratios (ICERs) that will be computed as the ratio of the incremental costs to provide WelTel SMS over usual care and incremental effects e.g. cost per additional mother-infant pairs that remain in PMTCT until infant is aged 24 months, cost averted infant HIV-infections at cessation of breastfeeding and cost per QALY.

A secondary analysis will also consider the incremental cost per averted deaths by bringing infants lost to follow up back and enabling treatment. Thus, we will determine ICER for cost per averted deaths. Deterministic sensitivity analysis will be to address uncertainty.

## Discussion

This trial provides an opportunity to test whether the WelTel SMS intervention is effective in improving retention in PMTCT care. Further, we will be able to determine whether the interactive WelTel text-messaging intervention, by engaging patients with the PMTCT clinic on a weekly basis, is a cost-effective way to improve retention in this critical stage of care, potentially helping to eliminate pediatric HIV infections.

## Abbreviations

AIDS, acquired immune deficiency syndrome; ANC, antenatal care; ART, antiretroviral therapy; ARVs, antiretroviral drugs; CEA, cost effectiveness analysis; CUA, cost utility analysis; HIV, human immunodeficiency virus; ICERs, incremental cost effectiveness ratios; IDIs, in-depth interviews; IREC, Institutional review and ethics committee; MCH, maternal and child health; MTCT, mother to child transmission; PIP, partners in prevention – Moi University; PLHIV, people living with HIV; PMTCT, prevention of mother to child transmission; QALYs, quality-adjusted life years; RCT, randomised clinical trial; RR, relative risk; SMS, short message service; UNAIDS, Joint United Nations programme on AIDS; WelTel SMS, Weekly mobile phone short message service
